# Cancer and Its Cure

**Published:** 1906-12-22

**Authors:** 


### Cancer and its Cure.

Mr. Edmund Owen did good service in his recent Bradshaw lecture when he made absolute the statement that, in the present state of medical and surgical knowledge and experience, the only way in which the cure of cancer can be obtained is by its prompt and thorough removal by operation. When a medical man has made up his mind that a growth is malignant and is capable of removal, he should at once urge the patient to consent to an immediate operation. No surgeon is justified in agreeing to any problematical scheme of treatment from a desire to lessen mental distress or to allay the dread which his patient may have of the use of the knife. This dread and mental distress are greatly overestimated, and are often due to the timidity of the medical man rather than of the patient himself. Very little repugnance is shown towards operative measures if the matter is carefully and kindly explained, the advantages put forward, and just conclusions drawn. The people whom it is difficult to persuade are those who have lost confidence in their medical adviser, or those who have been attended by a practitioner who is not himself sure of his own ground and, with a profession of zeal for surgical treatment, has yet a sneaking belief that the evil day may be postponed for the present by the trial, for a time, of non-operative measures. Such an attitude breeds doubt in the mind of the patient, and makes it difficult for him to come to the right decision.

No surgeon at the present time will undertake to *cure* a patient of cancer, though his policy in regard to the disease should be like that of Strafford to the constitution, a policy of *Thorough*. Yet he may succeed in arresting the progress of cancer so completely that it does not again show itself during the rest of the patient's life. Or again, he may secure a longer or shorter period of immunity after which recurrence takes place, or he may relieve the patient temporarily of the more distressing features of the malady by removing a fungating or bleeding mass. More than this he cannot do. It is still impossible to eradicate the disease by removing the cause, for the cause is as yet hidden, and it is not even known why removal of the tumour sometimes gives a long immunity and sometimes only a short one. The wisest surgeons are those who watch the progress of experimental and pathological research on cancer ready to avail themselves of every seeming advance; but to do this requires a rare combination of knowledge, opportunity, and judgment.

The frequent failure of surgery in dealing successfully with cancer unfortunately gives too many opportunities to try other methods of treatment, sometimes because recurrence has taken place, at other times because the disease has established itself from the beginning in a position beyond the reach of operation. After the surgeon, therefore, and often in place of him, come persons who promise a cure, even in advanced cases, by some special method of treatment. Such cancer-curers often deceive themselves into a belief that they actually possess the power they claim, and, being self-deceivers, they impart their confidence to their clients, whose last moments are thus inspired by the hope of recovery. An honest cancer-curer is thus useful in so far as he promotes euthanasia in an incurable disease. The diagnosis in the earlier stages of cancer is difficult and often erroneous, so that he can always point to cases which have apparently been successful in his hands, and he can account in a plausible manner for those which have been unsuccessful. But other cancer-curers exist of a much less reputable character, for, themselves undeceived, they deceive their patients, and by acting as quacks have done in all ages they enrich themselves by the sale at exorbitant prices of worthless, if harmless, preparations. Between the absolutely honest cancer-curer and the dishonest quack, exist a number of ignorant, well-meaning persons, who, beginning with honest doubt, and finding how easy it is to dupe the ignorant public, allow themselves to drift until they end in dishonesty. When the history of modern medicine comes to be published, it will be found that the treatment of cancer fills the position which has been occupied at various ages by the philosopher's stone, magnetism, lithontriptics, sympathetic cures, and crystal-gazing—subjects which have powerfully excited the popular imagination, and in connection with which there have been grains of scientific truth and hosts of impostors. The masses are, and must remain, credulous, for their education and mode of life do not call for extensive use of the critical faculties. Fortunately they have always been dominated ultimately by the judgment of those concerned with the advancement of knowledge. In time the cause of cancer will be discovered, and quacks and their remedies will then vanish from its treatment.

